# Effect of interaction with clowns on vital signs and non-verbal communication of hospitalized children

**DOI:** 10.1016/j.rppede.2016.02.011

**Published:** 2016

**Authors:** Pauline Lima Alcântara, Ariane Zonho Wogel, Maria Isabela Lobo Rossi, Isabela Rodrigues Neves, Ana Llonch Sabates, Ana Cláudia Puggina

**Affiliations:** a Faculdade de Medicina de Jundiaí (FMJ), Jundiaí, SP, Brazil; b Universidade Guarulhos (UnG), Guarulhos, SP, Brazil

**Keywords:** Non-verbal communication, Laughter therapy, Vital signs

## Abstract

**Objective:**

Compare the non-verbal communication of children before and during interaction with clowns and compare their vital signs before and after this interaction.

**Methods:**

Uncontrolled, intervention, cross-sectional, quantitative study with children admitted to a public university hospital. The intervention was performed by medical students dressed as clowns and included magic tricks, juggling, singing with the children, making soap bubbles and comedic performances. The intervention time was 20min. Vital signs were assessed in two measurements with an interval of 1min immediately before and after the interaction. Non-verbal communication was observed before and during the interaction using the Non-Verbal Communication Template Chart, a tool in which non-verbal behaviors are assessed as effective or ineffective in the interactions.

**Results:**

The sample consisted of 41 children with a mean age of 7.6±2.7 years; most were aged 7-11 years (n=23; 56%) and were males (n=26; 63.4%). There was a statistically significant difference in systolic and diastolic blood pressure, pain and non-verbal behavior of children with the intervention. Systolic and diastolic blood pressure increased and pain scales showed decreased scores.

**Conclusions:**

The playful interaction with clowns can be a therapeutic resource to minimize the effects of the stressing environment during the intervention, improve the children's emotional state and reduce the perception of pain.

## Resumo

**Objetivo:**

Comparar a comunicação não verbal das crianças antes e durante a interação com palhaços e comparar os sinais vitais antes e após essa interação.

**Métodos:**

Estudo intervenção não controlado, transversal, quantitativo, com crianças internadas em um hospital público universitário. A intervenção foi feita por alunos de medicina vestidos como palhaços e incluiu truques de mágica, malabarismo, canto com as crianças, bolhas de sabão e encenações cômicas. O tempo de intervenção foi de 20 minutos. Os sinais vitais foram avaliados em duas mensurações com um intervalo de um minuto imediatamente antes e após a interação. A comunicação não verbal foi observada antes e durante a interação por meio do Quadro de Modelos Não Verbais de Comunicação, instrumento em que os comportamentos não verbais são avaliados em efetivos ou ineficazes nas interações.

**Resultados:**

A amostra foi de 41 crianças com média de 7,6±2,7 anos, a maioria tinha entre 7-11 anos (n=23; 56%) e era do sexo masculino (n=26; 63,4%). Houve diferença estatisticamente significativa na pressão arterial sistólica e diastólica, na dor e nos comportamentos não verbais das crianças com a intervenção. As pressões arteriais sistólicas e diastólicas aumentaram e as escalas de dor mostraram diminuição na sua pontuação.

**Conclusões:**

A interação lúdica com palhaços pode ser um recurso terapêutico para minimizar os efeitos do ambiente estressor durante a intervenção, melhorar o estado emocional das crianças e diminuir a percepção de dor.

## Introduction

The joy therapy, also called laughter therapy or humor therapy, is a known therapeutic method since the 1960s. It was first introduced by the American physician Hunter Adams, also called “Patch Adams”, who since his medical student days already used the method in hospitals and schools. Joy is like a wave that propagates through all the nerves, organs, and glands of the whole body. Nothing is indifferent to laughter. Smiling and laughing are a universal language of communication that is expressed without words in the individual's face.^1^

The smile has great power and knowing how to smile is something important. Laughter is a uniquely human feature. It is a vital resistance mechanism and provides release of repressed feelings for coping with stress, suffering, or pain.^2^ It has the ability to reduce the harmful effects caused by stress in the body, because when a person laughs the parasympathetic system, through the enkephalins, acts on the immune system, increases the concentration of antibodies, and relieves the pain triggered by the sympathetic system.^3^

When laughing, the serum levels of cortisol decrease and the brain releases endorphins—substances that relieve pain and ensure the feeling of well-being. The heavy breathing increases the amount of air captured by the lungs and facilitates carbon dioxide output. Powerful analgesic, but also a producer of euphoria and sense of peace.^2^^,^ 4 Thus, the transmission of painful stimuli is inhibited and there is a “residual effect”.^4^

Smiling also has social benefits; it propagates from one individual to another, improves the bond between people, and clarifies interpersonal communication. Communication, as clear and objective as it may be, will always contain subjectivity because it involves human relationships, and the perception and interpretation of verbal and non-verbal messages happen through the sense organs: sight, touch, taste, smell, and hearing.^5^

Laughter is a non-verbal communication of well-being, but there are other signs that can be seen by a health professional. Noticing not only what the patient says verbally, but also the non-verbal cues, is essential to understand him completely, not only his pathology. The non-verbal body language has many messages for good observers^6^ by complementing, substituting, or contradicting the verbal speech. It is thus up to the professional to notice the signs and interpret them.^7^

Professionals should seek to understand the children in the holistic sense, meet their needs, abilities, and desires; it is evident that when the professional-patient relationship occurs efficiently, the care provided will be as beneficial as possible. Inevitably, the relationships that occur within the hospital environment will directly influence the child's treatment.^8^ Play is one of the needs of hospitalized children that needs to be met, because the physical, emotional, cognitive, and social development of children does not cease, even when they are ill.^9^

Moreover, play gives professionals a different experience with the children, not just dealing with disabilities and limitations. The clowns' performance can also provide socialization and interaction among children, which allows the creation of new social network; it acts as an enabling condition to get out of the social isolation that sometimes hospitalization provides. This fact may also be associated with the recovery condition.^8^ Playing also changes the environment in which the child is, making it closer to his/her reality. Thus, a free and disinterest recreation has therapeutic effect.^10^

In hospital settings, in which the admission process is usually an exhausting experience, children may associate it with fear, grief or sense of punishment. Among the many ways to reduce stress, improve bond, and understand the individual in its entirety, a playful interaction can be an effective strategy in this context. A ludic behavior provides beneficial effects, such as improving the clinical condition and reducing the anxiety and stress of the difficult time of hospital stay.^11^ In this sense, the ludic behavior emerges as an important resource to help children cope with the reality of hospitalization.

The above considerations show that among the ways to minimize the harmful effects of hospitalization is the playful activity, a strategy that helps the child to express their feelings. This study was done in order to better understand the effects of playful interaction of clowns in non-verbal communication and the physiological parameters of hospitalized children.

## Method

This is an uncontrolled, cross-sectional, interventional study, investigating quantitative variables, with vital signs and non-verbal communication as dependent variables.

The study was performed in the Pediatric unit of a public university hospital with 24 beds. Inclusion criteria were: (1) children aged 2-11 years; (2) admitted to the Pediatric unit; (3) hemodynamically stable; (4) awake and willing to participate. Children with intellectual and visual impairments that prevent them from identifying the design of faces pain scale or interact with the clowns were excluded.

The study development met the requirements of the Resolution 466/2012, in force in the country, on the ethics of research involving human subjects, and was approved by the Institutional Review Board of the Faculdade de Medicina de Jundiaí, Opinion No 840.408.

Data were collected from November 2014 to March 2015 and began with the approach of an investigator dressed in ordinary clothes: white coat, stethoscope, and clipboard. Informed Consent was explained to the guardians and Consent Term to the children over 6-year old. Children were told how the measurement of vital signs would be made, with approaches according to their developmental characteristics; the used equipment (stethoscope, sphygmomanometer, thermometer, and faces pain scale) were shown to them, and a careful approach was initiated with the children. Subsequently, a questionnaire of characterization was applied, which contained five questions about the child (age, number of siblings, sex, if attending school, and if the child does some physical activity), and vital signs were assessed (temperature, pulse, respiratory rate, blood pressure, and pain) in two time points with an interval of one minute. After that, the investigator thanked the child and said she would be in the room to make notes. A previous observation of non-verbal communication was carried out at this time.

The intervention was carried out through playful interaction of students, members of the “League of Joy” of the Medical School of Jundiaí. Ludic activity is regarded as all playful activities or any activity intended to produce pleasure during its practice; that is, having fun.^12^ The intervention included the work of volunteers from the League of Joy and aimed to minimize the stress of hospitalization through magic tricks, juggling, singing with children, soap bubbles, and comedic performances. The intervention time lasted 20min.

The non-verbal language during the intervention was recorded by the investigator who controlled the time. Subsequently, the same investigator assessed again the five vital signs of children in two measurements with 1min interval. After the measurement, the investigator thanked the parent accompanying the child, and the child himself, and departed.

Specifically, body temperature, blood pressure, respiratory and heart rate, pain, and non-verbal language were assessed. Respiratory rate was assessed by abdominal or chest observation and heart rate was measured by palpation at the radial artery and auscultation. For blood pressure measurement, an automatic digital blood pressure device Microlife Table Blue 3BTO-BP (Microlife^®^, Widnau, Switzerland) and the same brand cuffs suitable for arm circumference of the participants were used. This equipment is validated and certified by the British Society of Hypertension (BHS) and the Kidney and Hypertension Hospital of the Federal University of São Paulo. Temperature was recorded with a digital children's thermometer in the axilla, G-Tech with flexible tip-Urso (Accumed-Glicomed^®^, Rio de Janeiro, Brazil).

For pain assessment, considered the fifth vital sign,^13^ the faces pain scale that uses characters created by Maurício de Sousa, Cebolinha (chives) and Monica, expressing different emotional faces in each pain graduation. This scale was chosen because it is widely used in pain severity assessment in the Brazilian Pediatric population. The scale ranges from 0 to 4, with 0=no pain; 1=mild pain; 2=moderate pain; 3=severe pain; 4=excruciating pain.^14^ There were two measurements before and two measurements after the intervention. For analysis, however, an average was obtained before and after for each vital sign.

Non-verbal communication was analyzed using a Table of Nonverbal Models, which consists of a guideline for assessing non-verbal communication in different contexts; it is not a scale and does not have score.^7^ This instrument contains 14 items (posture, eye contact, furniture, clothing, facial expression, mannerism, voice volume, voice rhythm, energy level, interpersonal distance, touch, head, body posture, and paraverbal communication), which are assessed in effective or ineffective non-verbal behaviors in interactions. Effective behaviors are regarded as those that encourage speech or approach with others by showing acceptance and respect; ineffective behaviors are those that are likely to weaken the conversation and distance others from interaction. The interaction of children with clowns was evaluated using 7 of the 14 items in the Table of Nonverbal Models that best suited the context analyzed. The selected items refer to posture, eye contact, furniture, facial expression, energy level, head, and body posture. Non-verbal communication was considered as effective when the child's posture was relaxed and attentive, eye contact with regular frequency and average intensity in relation to the clowns; when the furniture or objects available were used to unite and not as barriers, the child was smiling, alert, nodded the head up and down (to say yes) and the body posture was focused on the clowns interacting with him/her. Ineffective non-verbal signals were observed when the child's posture was stiff and tense, challenging or absent look, the furniture or objects used as a barrier between people, the child's face was facing the other side, opposite to the clowns or expression less, when the child is apathetic, sleepy or restless during the interaction, shook his/her head sideways (to say no) and the posture was lateral or back turned to the clowns.^7^

In many studies assessing non-verbal communication, it is common that two observers do the assessment and compare opinions, precisely because the non-verbal decoding may be subjective. However, it is known that the more the individual feels observed, more this behavior is modulated and biased. Therefore, the option was for a single observer evaluation for the following reasons: let the children more comfortable to interact with the clowns, cause no embarrassment, and enable the therapeutic benefit of this study.

Quantitative data were entered into a database using Excel 2010 for later statistical analysis with the SPSS—Statistical Package for Social Science, version 23 (IBM^®^, Chicago, USA). Descriptive (absolute frequency, relative, mean and standard deviation) and inferential analysis were performed. Kolmogorov-Smirnov test was used for data normality and parametric or non-parametric tests were used according to distribution. For mean comparisons before and after vital signs, Wilcoxon and Student's *t* tests were used. McNemar test was used to compare the change or retention of effective or ineffective non-verbal behaviors assessed before and during the intervention. This test compares the differences between two samples and identifies changes in the observation of a variable. The sample size was based on the number of children in studies of ludic behavior identified in the literature.

## Results

The total sample consisted of 41 children, mean age of 7.6±2.7 years, most aged between 7 and 11 years (n=23; 56%), male (n=26; 63.4%), one or two siblings (n=28; 68.3%), attending school (n=38; 92.7%), do physical activity (n=32; 78%) (Table 1).

**Table 1:** Characteristics of children. Jundiaí, 2014-2015.

Variable	n	%
*Age*		
3-6 years	18	43.9
7-11 years	23	56.1

*Sex*		
Female	15	36.6
Male	26	63.4

*Number of siblings*		
0	5	12.2
1	15	36.6
2	13	31.7
≥3	8	19.5

*Attending school*		
Yes	38	92.7
No	3	7.3

*Physical activity*		
Yes	32	78.0
No	3	7.3

There were significant changes between the means before and after intervention when comparing systolic and diastolic blood pressures and pain. After the playful interaction, there were increase in systolic and diastolic blood pressures and decreased pain (Table 2). On average, systolic blood pressure increased from 112×71 to 75×117 and pain from 1.1 to 0.6, apparently not clinically significant. However, it indicates physiological and beneficial changes with the playful interaction of children with clowns. That is, this result shows that there was a relationship between blood pressure, pain, and playful activity, as the children showed a positive emotional response evidenced by an increase in energy level, smiling facial expression, and active participation in games with the clowns (Tables 3 and 4).

**Table 2:** Mean comparison before and after vital signs assessment. Jundiaí 2014-2015.

Vital signs	Before			After		*p*-value
	Mean	SD		Mean	SD	
Respiratory rate	26.2	8.0		25.9	6.8	0.944
Pulse	93.5	17.5		95.0	16.6	0.574^a^
Systolic blood pressure	112.2	13.0		116.7	14.9	0.047
Diastolic blood pressure	71.0	11.7		75.0	15.7	0.040
Temperature	36.3	0.5		36.3	0.6	0.712^a^
Pain	1.1	1.2		0.6	1.0	0.001

Wilcoxon test.

a Student's *t* test.

**Table 3:** Comparison of change or permanence of effective or ineffective non-verbal behaviors assessed before and during the intervention. Jundiaí 2014-2015.

Non-verbal behavior	n	%	*p*-value
*Posture*			**0.004**
Relaxed but attentive before and during^a^	28	68.3	
Relaxed but attentive before and rigid during^b^	0	0.0	
Rigid before and relaxed but attentive during^c^	9	22.0	
Rigid before and during^d^	4	9.8	

*Eye contact*			**0.002**
Regular, average before and during^a^	27	65.9	
Regular, average and absent before, challenging during^b^	0	0.0	
Absent, challenging before and regular, average during^c^	10	24.4	
Absent, challenging before and during^d^	4	9.8	

*Furniture*			**0.016**
Used to interact before and during^a^	32	78.0	
Used to interact before and used as a barrier during^b^	0	0.0	
Used as a barrier before and used to interact during^c^	7	17.1	
Used as a barrier before and during^d^	2	4.9	

*Facial expression*			**<0.001**
Smiley, shows feelings before and during^a^	24	58.5	
Smiley, shows feelings before and turns his face to the other side or expressionless during^b^	0	0.0	
Face turned to the other side or expressionless before and smiley, shows feelings during^c^	14	34.1	
Face turned to the other side or expressionless before and during^d^	3	7.3	

McNemar test. Assessed situations:

a Effective remains effective.

b Effective becomes ineffective.

c Ineffective becomes effective.

d Ineffective remains ineffective. In situations ^a^ and ^d^ behaviors do not change with the intervention. In situations ^b^ and ^c^ behaviors are changed with the intervention.

**Table 4:** Comparison of change or permanence of effective or ineffective non-verbal behaviors assessed before and during the intervention. Jundiaí 2014-2015.

Non-verbal behavior	n	%	*p*-value
*Nível de energia*			**<0.001**
Alert before and during^a^	16	39.0	
Alert before and lethargic, sleepy, cyclic or restless during^b^	0	0.0	
Lethargic, sleepy, cyclic or restless before and alert during^c^	19	46.3	
Lethargic, sleepy, cyclic or restless before and alert during^d^	6	14.6	

*Head*			0.125
Nodded before and during^a^	30	73.2	
Nodded before and shook the head negatively during^b^	0	0.0	
Shook the head negatively before and nodded during^c^	4	9.8	
Shook the head negatively before and during^d^	7	17.1	

*Body posture*			**0.004**
Facing the person before and during^a^	29	70.7	
Facing the person before and sideways or back turned during^b^	0	0.0	
Sideways or back turned before and facing the person during^c^	9	22.0	
Sideways or back turned before and after^d^	3	7.3	

McNemar test. Assessed situations:

a Effective remains effective.

b Effective becomes ineffective.

c Ineffective becomes effective.

d Ineffective remains ineffective. In situations ^a^ and ^d^ behaviors do not change with the intervention. In situations ^b^ and ^c^ behaviors are changed with the intervention.

There were statistically significant changes in the comparison of non-verbal behaviors before and during the intervention regarding posture, eye contact, use of furniture or objects in the interaction, facial expression, energy level, and body posture; that is, in six of the seven behaviors observed (Tables 3 and 4).

The non-verbal behavior changes were all ineffective to effective behaviors during the intervention; that is, the previous rigid posture became relaxed and attentive (n=9, 22.0%). The absent and challenging eye contact became regular and average (n=10; 24.4%). An absent or fixed gaze is uncomfortable and can be invasive in relationships; a frequent look (regular) and with proper intensity (average) is comfortable and facilitates interaction with others. Furniture and objects previously used as a barrier (e.g., sheet over the head or covering much of the body) were used to interact or were removed (n=7; 17.1%). The child's face, turned predominantly to a side of the room or expressionless, changed to a smiling face in the presence of clowns and showed feelings (n=14; 34.1%). The energy level, previously lethargic, sleepy, cyclic or restless, became alert (n=19; 46.3%). Finally, the child's body posture, initially observed on sideways or back turned, faced the clowns, showed openness and acceptance in interpersonal relationships (n=9; 22.0%) (Tables 3 and 4).

The non-verbal behavioral changes found with the intervention show the effectiveness of playful activities with clowns as a therapeutic resource. In general, the children were more relaxed, open, and smiley. The intervention was able to modify the initial context.

## Discussion

In this study, the investigators sought to make a close contact, humanized, and individual with children, as the visits were made inside the room where they were staying and the games flowed differently in each visit. The importance of this form of playful interaction has been shown in a study conducted in Australia, in which videoconference was held to promote interaction between clowns from Royal Children's Hospital with children hospitalized or at home. The experience has shown that interaction between clowns and institutionalized children via videoconference is technically feasible and practical. However, it would be necessary to make individual games for each child, something that would be easier personally. In this scenario, the online interaction was more limited, but it is no longer an option.^15^

Literature studies^16^^,^ 17 report convergent results with those found by us. The playful interaction of children with clowns, as shown in this study, was an effective strategy of redirecting the energy of children to positive and beneficial feelings. The non-verbal behavioral changes during the intervention showed that children become more relaxed, attentive, and smiley. A study was performed in Portugal with 70 children, aged 5-12 years, divided into two groups for preoperative outpatient monitoring. In one group, the children were monitored in the room by their parents and two clowns; in the other group, they were monitored only by their parents. The Child Surgery Worries Questionnaire was used to describe the patients' distress. Children monitored by clowns were less worried with hospitalization and medical procedures, less concerned with the disease itself, and felt happier and calmer compared to the other group.^16^

In another case-control study, 60 children, aged 6-10 years, who were scheduled for surgery, were recruited. Of these, 30 would receive a visit from two clowns before surgery (case group) and 30 would not receive it (control group). Anxiety was measured using the following scales: State Trait Inventory Anxiety for Children, Community-Campus Partnerships for Health, and Faces Pain Scale, after the performance of clowns and up to seven days after surgery. Both groups showed increased anxiety, but in the group of children undergoing clown intervention, the increased anxiety was less important.^17^

Similar results to the present study regarding blood pressure were found in a study performed in Japan. Seventeen apparently healthy adults, aged 23-42 years, watched a 30-min comedy (experimental) and a documentary (control) on different days. Heart rate and blood pressure increased significantly while the subjects watched comedy, there were no such changes during the documentary.^18^ Laughter and joy cause excitement and well-being.

The relationship between humor and pain was studied with 80 participants, aged 18-44 years. In this investigation, the cold stimulation was made with water maintained at 1ºC by an immersion chiller and circulation by an underwater mixer. On top of the water there was an arm resting place on which the participant held the left arm. Participants were divided into four groups of 20. Group 1 watched a humorous film, Group 2 watched a repulsive film, Group 3 watched a neutral film, and Group 4 had no film to watch. State-Trait Anxiety Inventory, Humor Questionnaire, measurement items for self-efficacy for pain control, visual analog scale for anxiety, and a post-experiment questionnaire were used. Pain tolerance significantly increased with humor.^19^

In addition to the behavioral and physiological effects, the benefits of interacting with clowns are not restricted to patients; family and professionals seem to also benefit.^20^^-^ 22 Literature studies confirm this finding, and this perception was also corroborated in the current study, although not documented, as both the team and health professionals verbalize their praise for the research initiative and to the students members of the League of Joy. Interaction with clowns interferes in a whole context in which the child is placed. An example of this was found in a study performed in Germany, in which one of the objectives was to evaluate the performance of clowns by the parents of hospitalized children and by the hospital staff. The study included 37 parents and 43 staff, and a satisfaction scale and monitoring at the acting field were applied. Both the parents and the staff reported that they and patients benefit from the intervention.^20^

An ethnographic study was performed to assess principles, values, and methodology of the Associação Operação Nariz Vermelho (Red Nose Operation Association) during visits to hospitalized children. The results show a strong relationship of empathy and complicity between the Clown Doctors and the children, as well as a strong sense of belonging, on the part of artists, to the hospital community, visible in the relationship established with professionals and in delivering a quality care that provides well-being and joy. This sense of sharing and creating ties extends also to the children's relatives, who act as channels of communication between the relationship Clown Doctor and the child.^21^

To show that the changes are not perceived only by those attending the presentation, a study investigated something different in this regard. The expectations of Pediatric professionals were analyzed (n=34) through a semi-structured interview about the advantages and disadvantages of the presence of clowns among children and teens, even before the intervention. Data revealed a wide opening to the presence of artists, pointing them as potential minimizers of the hospitalization and treatment emotional impact and highlighted their contribution to the humanization of care and demystification of health professionals. However, the reported disadvantages were panic or fear of clown by some children, little receptivity due to suffering, and resistance to the presence of clowns by adolescents due to the childishness involved.^22^

Finally, the survey of this intervention disadvantages^22^ brings to discussion the recognition of limitations regarding the playful interaction with clowns, such as the fear of clown arising mainly from fantasies, which makes it essential that professionals involved in playful activities with clowns show sensitivity, common sense, and respect for children and their negative reactions (crying, screaming, refusal to play with clown) in order to be really beneficial and therapeutic.

Despite its limitations, such as observational bias (a single observer assessed the behavior and applied the non-verbal behavior scale), option for the application of a non-validated instrument, and measurement bias (lack of vital signs continuous measurement), the study has outcomes indicating that the playful interaction with clowns can be a therapeutic resource to minimize the effects of the stressor environment during the intervention, improve the emotional status of children, and reduce pain perception.
